# Epigenetic regulation of glucosinolate biosynthesis sees the light of day

**DOI:** 10.1093/plphys/kiae343

**Published:** 2024-06-14

**Authors:** Moona Rahikainen

**Affiliations:** Assistant Features Editor, Plant Physiology, American Society of Plant Biologists; Organismal and Evolutionary Biology Research Program, Faculty of Biological and Environmental Sciences, University of Helsinki, FI-00014 Helsinki, Finland

Glucosinolates (GSL) are specialized defense metabolites synthesized by species of the order Capparales, including the economically important Brassicaceae crops such as oil seed rape, cabbages, and kales. Upon tissue damage, GSLs stored in the vacuole are degraded by myrosinases together with specifier proteins. The breakdown products include thiocyanates, isothiocyanates, nitrile, and epithionitriles, which are toxic to pathogens and herbivores (Wittstock and Burow 2010). In addition to their role as defense compounds, various GSLs have health promoting effects and contribute to the bitter and pungent taste of Brassicaceae vegetables (Bell et al. 2018; Wu et al. 2023). A total of 137 different GSL structures have been identified in various species and divided into 3 subcategories, aliphatic glucosinolates, indole glucosinolates, and benzyl glucosinolates, according to the amino acid precursor used in their biosynthesis (Blazevic et al. 2020). The GSL biosynthetic pathways have been extensively studied in the model plant *Arabidopsis thaliana* (hereafter Arabidopsis). GSL biosynthesis is composed of 3 phases that include chain elongation, core structure formation, and side chain modification that results in a variety of different compounds with nitrogen and sulfur containing core structure and highly variable side chain (Blazevic et al. 2020).

Plant GSL content undergoes diurnal fluctuations, and several GSL biosynthesis enzymes and transcription factors show light-dependent expression patterns (Huseby et al. 2013). In this issue of *Plant Physiology*, Choi et al. (2024) identify ELONGATED HYPOCOTYL 5 (HY5) transcription factor together with HISTONE DEACETYLESE 9 (HDA9) as regulators of light-induced changes in GSL content in Arabidopsis (Fig. 1). The authors demonstrate diurnal fluctuation in both aliphatic and indole glucosinolates under long day conditions and show that total GSL content peaks 4 hours into the light period and declines towards the end of light period. Corresponding fluctuations were observed in the expression of genes in the aliphatic glucosinolate pathway, whereas the genes of the indole glucosinolate pathway showed more complex expression patterns. HY5, a bZIP-family master regulator of light-dependent responses in Arabidopsis, was previously shown to control light-dependent sulfur assimilation, a process coregulated with GSL biosynthesis (Huseby et al. 2013). Choi et al. (2024) investigated the involvement of HY5 in GSL content under diurnal light period and observed higher levels of aliphatic glucosinolates in the null *hy5-215* mutant (*hy5*) compared with wild type in most timepoints through the diurnal cycle. In contrast, the levels of indole glucosinolates showed an opposite trend. Further transcriptomic profiling and gene ontology analysis of *hy5* mutant identified GSL biosynthesis as a top category among the enriched biological processes. Closer examination of GSL biosynthesis genes with qPCR suggested that HY5 functions as a suppressor of aliphatic glucosinolate biosynthesis.

**Figure 1.**
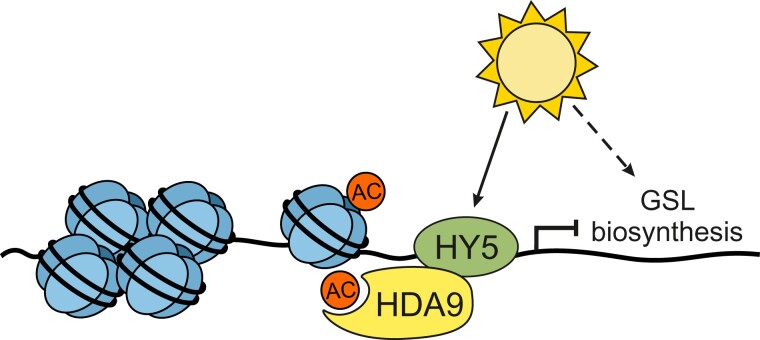
GSL biosynthesis is activated under light. HY5 recruits HDA9 to specific genetic regions to modulate plant GSL content. Histone deacetylation induces chromatin condensation and suppresses the expression of genes in the aliphatic GSL pathway.

GSL biosynthesis enzymes are transcriptionally regulated by MYB transcription factors (Hirai et al. 2007). MYB29, controlling the aliphatic GSL pathway, was found to be upregulated in *hy5* (Choi et al. 2024). Using chromatin immunoprecipitation (ChIP) with qPCR and electrophoretic mobility shift assay, Choi et al. (2024) confirmed that HY5 binds to a G-box motif in the promoters of *MYB29* and *ISOPROPYLMALATE DEHYDROGENASE 1* (*IMD1*, involved in the chain elongation phase). HY5 was previously demonstrated to interact with HISTONE DEACETYLASE 9 (HDA9) for epigenetic regulation of HY5 target genes (Yang et al. 2020; Chu et al. 2022; Yang et al. 2023). In the current research, the authors used multiple complementary approaches to confirm that HY5 interacts with HDA9 and employed ChIP-qPCR to demonstrate that HDA9 also binds to *MYB29* and *IMD1* promoters. In line with the hypothesis that HY5 and HDA9 together regulate GSL biosynthesis, the Arabidopsis *hda9* mutant showed higher daytime GSL content and upregulation of aliphatic glucosinolate pathway genes compared with wild-type plants.

Histone 3 (H3) acetylation sites H3K9ac and H3K27ac were previously identified as targets of HDA9 (Chu et al. 2022). Choi et al. (2024) conducted ChIP assay with specific antibodies to compare H3 acetylation in wild type and *hy5* mutant. H3K9 acetylation was higher in the genomic regions of *MYB29* and *IMD1* in *hy5*, whereas no differences were observed in H3K27 acetylation. In line with the observed increase in H3K9 acetylation, the expression of *MYB29* and *IMD1* was upregulated in *hy5*, *hda9-1*, and *hy5/hda9-1* mutants compared with wild type. The HY5-HDA9 module was previously shown to regulate flowering time, salt tolerance, and autophagy in relation to light in Arabidopsis (Yang et al. 2020; Chu et al. 2022; Yang et al. 2023). The findings of this research extend the role of the HY5-HDA9 module as a central epigenetic modulator of light-dependent processes to include GSL biosynthesis. Moreover, the results support a mechanism where histone deacetylases are recruited to chromatin by DNA binding transcription factors to suppress the expression of specific genes.

Altogether, the work by Choi et al. (2024) presents an epigenetic regulatory mechanism that dampens the light-induced production of aliphatic GSLs. The characterization of the light-dependent positive regulators of GSL biosynthesis remains an open question for further research. Moreover, the observed diurnal fluctuations in GSL content also require regulated turnover of GSL in intact plant tissues. These mechanisms and their regulation are currently poorly understood (Wittstock and Burow 2010; Blazevic et al. 2020). GSLs are an interesting target for breeding in economically important Brassicaceae crops, since they have nutritional effects, a key role in plant defense, and they affect the sensory attributes of the vegetables (Wittstock and Burow 2010; Wu et al. 2023). In light of their research findings, Choi et al. (2024) propose circadian clock components as possible breeding targets to improve the quality of Brassicaceae crops.

## Data Availability

No new data was generated or analysed for this article.
